# The Effect of Different Intensities of Treadmill Exercise on Cognitive Function Deficit Following a Severe Controlled Cortical Impact in Rats

**DOI:** 10.3390/ijms141121598

**Published:** 2013-10-31

**Authors:** Xiafeng Shen, Aiping Li, Yuling Zhang, XiaoMin Dong, Tian Shan, Yi Wu, Jie Jia, Yongshan Hu

**Affiliations:** 1 Department of Rehabilitation, Huashan Hospital, Fudan University, Shanghai 200040, China; E-Mails: shenxiafeng@aliyun.com (X.S.); zhangyuling1982@hotmail.com (Y.Z.); shantian2008@hotmail.com (T.S.); wuyi4000@163.com (Y.W.); shannonjj@126.com (J.J.); drhuys@sina.com (Y.H.); 2 Rehabilitation Medicine Center, Nanjing Military Region Sanatorium of Hangzhou, Hangzhou 310007, Zhejiang, China; E-Mail: dongxiaomin2013@163.com; 3 Department of Rehabilitation, Shanghai Yangpu District Geriatric Hospital, Shanghai 200090, China

**Keywords:** controlled cortical impact, treadmill running, spatial memory, intensity, BDNF

## Abstract

Exercise has been proposed for the treatment of traumatic brain injury (TBI). However, the proper intensity of exercise in the early phase following a severe TBI is largely unknown. To compare two different treadmill exercise intensities on the cognitive function following a severe TBI in its early phase, rats experienced a controlled cortical impact (CCI) and were forced to treadmill exercise for 14 days. The results revealed that the rats in the low intensity exercise group had a shorter latency to locate a platform and a significantly better improvement in spatial memory in the Morris water maze (MWM) compared to the control group (*p* < 0.05). The high intensity exercise group showed a longer latency and a mild improvement in spatial memory compared to the control group rats in the MWM; however, this difference was not statistically significant (*p* > 0.05). The brain-derived neurotrophic factor (BDNF) and p-CREB protein levels in the contralateral hippocampus were increased significantly in the low intensity exercise group. Our results suggest that 2 weeks of low intensity of treadmill exercise is beneficial for improving cognitive function and increasing hippocampal BDNF expression after a severe TBI in its early phase.

## 1. Introduction

Many studies have reported that physical exercise is the most effective method to improve cognitive function and brain health. In addition, a number of human studies have shown that exercise is associated with delayed memory decline, reduced incidence of dementia, reduced depression and anxiety, improved cognitive function, including executive function and information processing speed, and reduced hippocampal atrophy in ageing humans [1–5]. Consistent with human research, animal studies demonstrate that exercise can facilitate neuronal plasticity, improve cognitive function, and promote recovery in normal or traumatic brain injury (TBI) rodent models [6,7].

TBI is known to result in deficits in spatial learning and memory. After TBI, cognitive impairments such as problems with memory, orientation, attention, executive functions and problem-solving are often prominent and long-lasting [8,9]. These cognitive deficits affect the recovery of motor function and affect daily life. Both the lateral fluid percussion injury (FPI) and controlled cortical impact (CCI) models of TBI result in cognitive impairments [7,10]. Cognitive dysfunctions are frequently associated with impaired hippocampal function [11]. Pathological and molecular systems associated with cognitive changes in the hippocampus have also been observed in CCI models in animals [12,13]. Massive neuronal death in the hippocampus, particularly in the CA2 and CA3 subfields, is the main cause of cognitive dysfunction following a TBI [14].

Some rehabilitation specialists believe that early rehabilitation intervention is beneficial in the treatment of TBI, and it has been applied in the ICU in some TBI centers [15]. Clinical studies have demonstrated that early rehabilitation can improve motor and cognitive function in TBI patients [15,16]; however, there is no consensus on the efficacy of early rehabilitation and proper intensity of exercise in severe TBI patients [15]. Some previous animal studies have demonstrated that exercise can normalise cognitive deficit following TBI [17,18]. Animal studies have also provided insight into the effect of exercise on brain function on a biological or molecular level following TBI [18]. For example, exercise suppresses neuronal and hippocampal apoptosis, inhibits astrocytic reactions, increases neural stem cell proliferation and neurogenesis, attenuates proteasome activity, reduces the protein levels of myelin-associated glycoprotein (MAG) and Nogo-A, reduces free radicals, and upregulates Na(+),K(+)-ATPase enzyme activity in the brain [17,19–24]. Our recent data also showed that an early initiation of treadmill exercise could improve cognitive performance following severe TBI (unpublished results). However, not all animal studies have consistently demonstrated that exercise improves cognitive function following TBI. Some studies have indicated that early exercise has no effect on or even reduces cognitive function [10,18,25], which suggests that different conclusions regarding early rehabilitation after TBI may depend on factors such as exercise intensity, exercise frequency, type of exercise, or other variables that have not yet been confirmed. With the definitions of rehabilitation intervention time and exercise type, the intensity of exercise should be a crucial variable that affects the treatment outcome after a TBI. Different intensities of exercise may induce different effects on learning and memory at an early phase following TBI. We hypothesised that low intensity of treadmill exercise after injury may be beneficial for the recovery of function after a severe TBI. To examine this hypothesis, rats experienced surgery to induce severe CCI injuries and were then forced to exercise on a treadmill with two different intensities beginning 24 h after the injury.

Synapsin I plays a role in synaptic vesicle clustering and neurotransmitter release [26,27]. The transcription factor cyclic AMP response element binding protein (CREB) is a transcription factors in the CNS and is involved in cell survival in the CNS and spatial learning [28,29]. CREB can be modulated by brain-derived neurotrophic factor (BDNF) [30].

Some types of molecular signaling, such as BDNF, synapsin I, and insulin-like growth factor 1 (IGF-1), may be involved with regulating the mechanisms of exercise-enhanced cognitive functions [18,31,32]. BDNF is the most abundant neurotrophic factor. BDNF has long been thought to be a potent neurotrophic factor that facilitates the growth, proliferation and differentiation of hippocampal neurons, and it is closely linked to cognition [33–35]; therefore, BDNF, synapsin I, and CREB proteins in the brain were also evaluated.

## 2. Results and Discussion

### 2.1. Effects of Treadmill Exercise on Weight Loss after CCI

**Figure 1:**
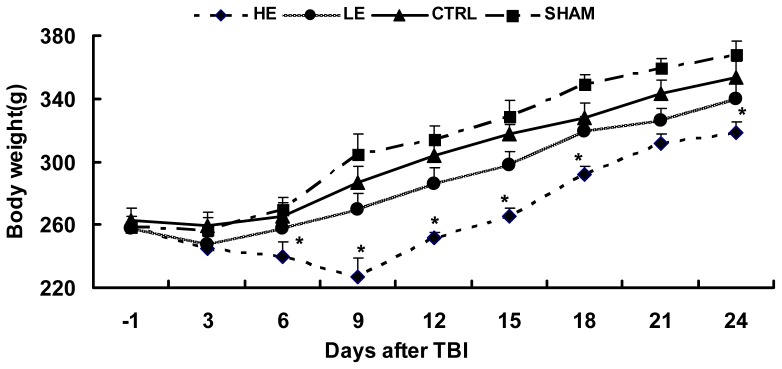
The effects of treadmill exercise on weight loss following TBI. No differences in initial, baseline body weights among the treatment groups were observed. All animals except HE group displayed a mild weight loss and recovered at day 6 after TBI. The LE group showed a mild weight loss than CTRL, this difference was not reach statistically significant (*p* > 0.05). The HE group showed a significant weight loss, recovered after 9 days after TBI and there were significant different between HE group and CTRL group. The data are the mean times ± standard errors of the mean (SEM). *p < 0.05 compared to the control.

All animals, except the high intensity exercise (HE) group, displayed a mild weight loss during the first 6 days post-injury and recovered quickly. The weight of the low intensity exercise (LE) group was lower than control (CTRL) group; this difference was not statistically significant (*p* > 0.05). The HE group showed a significant weight loss, and recovered gradually to exceed the pre-injury weights. There were significant differences between the HE group and CTRL group (Figure 1).

### 2.2. Neurologic Deficit Scores in Each Group

**Figure 2:**
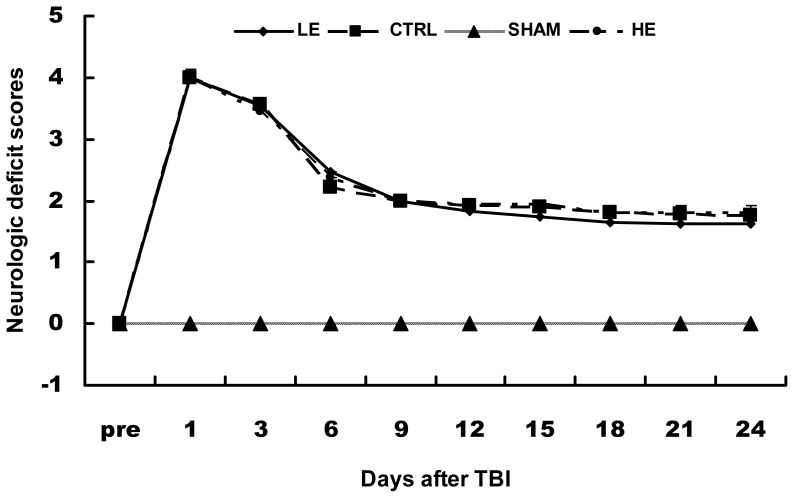
The effects of treadmill exercise on neurological deficit scores. The TBI rats had neurological deficit scores that decreased quickly within the first 6 days, and decreased more slowly at later time points. The TBI rats could not recover to normal at day 24.

All animals, except sham (SHAM) group, showed motor impairments after recovery from anesthesia. The SHAM group demonstrated no neurological deficit at any of the time points measured. The TBI rats exhibited neurological dysfunction with the first measurement at day 1. The TBI rats significantly improved neurological scores within the first 6 days post-injury, and recovered slowly until the last measured time at day 24. None of the TBI rats returned to baseline by the last day of testing. There was no statistic difference among the TBI groups (Figure 2).

### 2.3. Spatial Learning and Memory Performance

**Figure 3:**
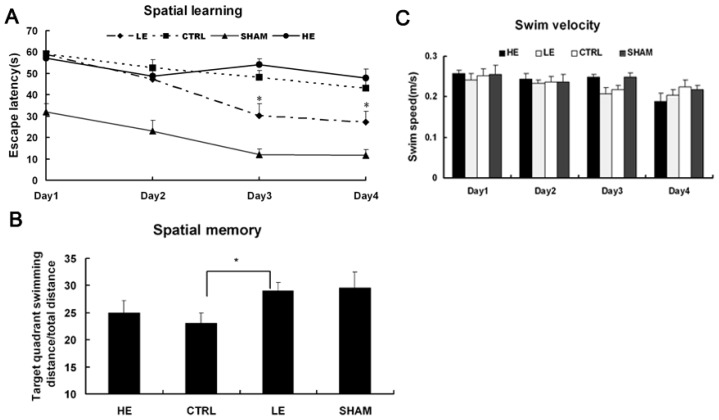
(**A**) The latency in the MWM task following TBI. The test began on day 21 post-injury and demonstrates that the control rats showed a significantly longer latency on days 3 and 4 of training (days 23 and 24 post-TBI, respectively) compared to the LE group (*p* < 0.05). The arrival time to the platform increased in the HE exercise group compared to the control group, but this difference was not statistically significant (*p* > 0.05). The data are the mean times ± standard errors of the mean (SEM). *p < 0.05 compared to the control; (**B**) A probe test was used to determine spatial memory on day 25, post-TBI. The hidden platform was removed, and the percentage of the total time spent in the target quadrant was measured. The LE group rats showed improved memory compared to the CTRL group indicated by significantly increased time swimming in the target quadrant (*p* < 0.05). The HE group spent a longer time swimming in the target quadrant compared to the CTRL group, but the difference was not statistically significant (*p* > 0.05). *p < 0.05 compared to the control; (**C**) The swim speed in the MWM task following TBI. No significant difference in swim speed was observed between LE and CTRL or HE and CTRL.

TBI is known to result in deficits in spatial learning and memory. The assessment of spatial learning revealed that both the LE and CTRL groups demonstrated time-dependent improvements in latency to locate the submerged platform. The rats in the LE group showed a significantly shorter latency on days 3 and 4 of training (days 23 and 24 post-TBI, respectively) compared to the control and HE groups (*p* < 0.05) (Figure 3A). The HE group showed a longer latency compared to the control group on days 3 and 4 of training (days 23 and 24 post-TBI, respectively); this difference was not statistically significant (*p* > 0.05). The LE group spent a significantly longer time in the target quadrant compared to the CTRL group (*p* < 0.05). The HE group spent a slightly longer time in the target quadrant compared to the CTRL group; this difference was not statistically significant (*p* > 0.05). There were no retention times of the target quadrant difference between the HE group and LE groups (*p* > 0.05) (Figure 3B). The result revealed that the swim speeds were similar in four groups (Figure 3C); in addition, the swim velocity did not influence the escape latency.

### 2.4. Effects of Treadmill Exercise on Hippocampal BDNF Protein

**Figure 4:**
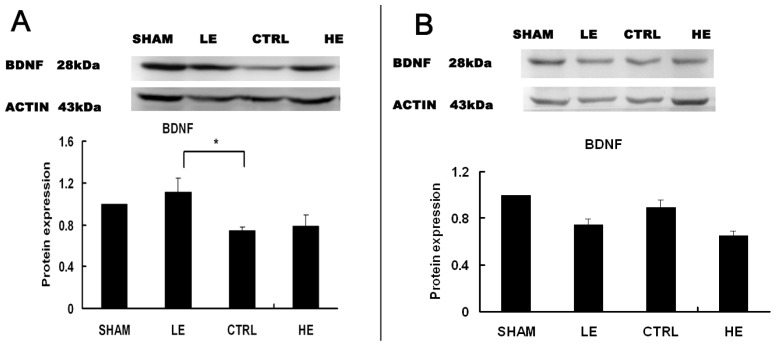
Effects of treadmill exercise on hippocampal BDNF. (**A**) The levels of BDNF were significantly increased in the low intensity exercise group (LE) compared with the control group (CTRL) in the contralateral hippocampus. The levels of BDNF were similar in the HE and CTRL groups (*p* > 0.05). *p < 0.05 compared to the control group; (**B**) The levels of BDNF were increased in the control group (CTRL) compared to the low intensity exercise group (LE) or high intensity exercise group (HE) in the ipsilateral hippocampus; there was no statistically significant difference between the three groups (*p* > 0.05). The data are represented as the means ± SEM.

BDNF was significantly higher in the LE group compared to the CTRL group in the contralateral hippocampus (*p* < 0.05) (Figure 4A). BDNF was similar in the LE and HE group compared to the CTRL group in the ipsilateral hippocampus (Figure 4B).

### 2.5. Effects of Treadmill Exercise on Hippocampal Synapsin I Protein

**Figure 5:**
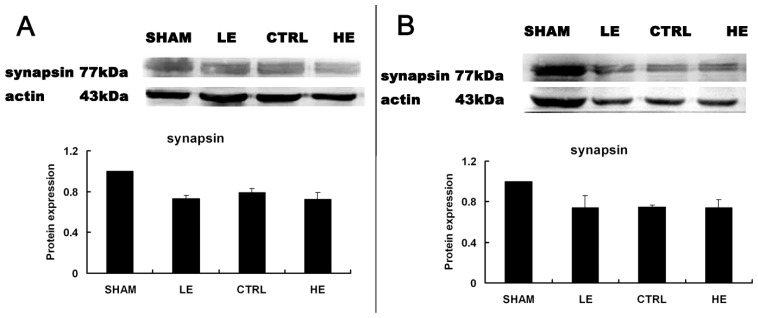
Effects of treadmill exercise on hippocampal synapsin I. (**A**)The levels of synapsin I were similar in the LE and HE group compared to the CTRL group in the contralateral hippocampus(*p* > 0.05); (**B**) The levels of synapsin I were similar in the LE, CTRL and HE group in the ipsilateral hippocampus (*p* > 0.05). The data are represented as the means ± SEM.

Synapsin I was similar in the LE and HE group compared to the CTRL group in the contralateral hippocampus (Figure 5A) or ipsilateral hippocampus (Figure 5B) (*p* > 0.05).

### 2.6. Effects of Treadmill Exercise on Hippocampal CREB Protein

**Figure 6:**
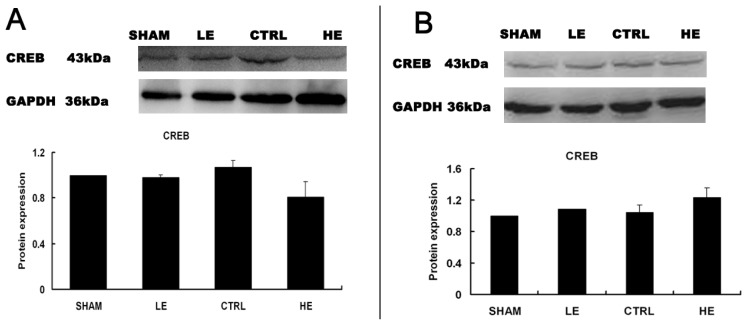
Effects of treadmill exercise on hippocampal CREB. The levels of CREB were lower in the LE and HE group compared to the CTRL group in the contralateral hippocampus (**A**); The levels of CREB were slightly increased in the HE group compared with the CTRL and LE group in the ipsilateral hippocampus, but these values also did not reach statistical significance (**B**) (*p* > 0.05). The data are represented as the means ± SEM.

No significant difference in CREB was observed between LE and CTRL or HE and CTRL in the contralateral hippocampus (Figure 6A) (*p* > 0.05). The levels of CREB were slightly increased in the HE group compared with the CTRL and LE group in the ipsilateral hippocampus, but these values also did not reach statistical significance (Figure 6B) (*p* > 0.05).

### 2.7. Effects of Treadmill Exercise on Hippocampal Phosphorylated CREB Protein

**Figure 7:**
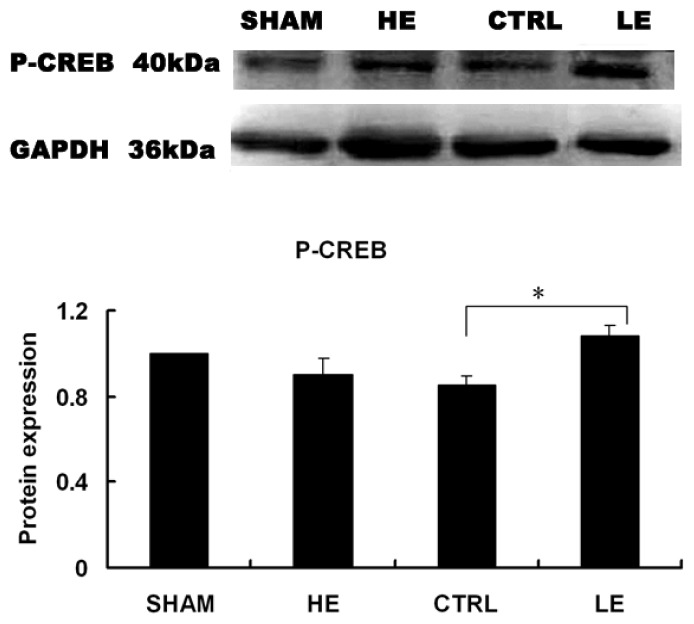
Effects of treadmill exercise on hippocampal p-CREB. The levels of p-CREB were significantly increased in the LE compared with the CTRL in the contralateral hippocampus (*p* > 0.05). * *p* < 0.05 compared to the control group.

The levels of p-CREB were significantly increased in the low intensity exercise (LE) group compared with the control (CTRL) group in the contralateral hippocampus (Figure 7). The levels of p-CREB were similar in the HE and CTRL groups (*p* > 0.05). * *p* < 0.05 compared to the control group.

### 2.8. Discussion

To our knowledge, our study is one of the very few studies involving the effect of different exercise intensities on cognitive function following severe TBI in its early phase. The present results reveal that the LE group showed similar neurological score compared to the CTRL group. However, the LE group demonstrated a shorter latency and spent significantly more time swimming in the target quadrant compared to the CTRL group. The data showed that the early initiation of low intensity treadmill exercise could improve cognitive performance following severe TBI. The data also revealed that the BDNF and p-CREB levels of the LE group in the contralateral hippocampus are upregulated, suggesting that the improvement in learning and memory is associated with the upregulation of BDNF in the contralateral hippocampus. Here, we found increased hippocampal p-CREB in low intensity trained rats. Therefore, it is possible to suppose that the increased BDNF levels may be associated with the activation of the ERK/CREB/BDNF signaling pathway. Studies have shown that exercise improves cognitive function and may contribute to an increase in BDNF within the hippocampus [36]. The increase of BDNF expression is consistent with a previously conducted study of forced or volunteer exercise following FPI in rats [7,10].

Griesbach *et al*. [18,37] reported that improvement in cognitive function was associated with increasing the expression of BDNF and synapsin I within the hippocampus after exercise in rats with TBI. In this experiment, we found that the levels of synapsin I and CREB were similar in the LE and HE group compared to the CTRL group in hippocampus. One possible cause for this difference may be related to the injury severity, modality of the exercise, intensity of the exercise, and duration of the exercise. However, an alternative interpretation can be that in LE group the time point for killing rats already passed the synapsin I and CREB expression positive phase.

Our results demonstrate that high intensity exercise on a treadmill following CCI injury does not attenuate cognitive deficits. This result suggested that high intensity treadmill exercise is not beneficial for cognitive function after early phase TBI. The result is similar to a previous study in an early phase FPI injury of moderate severity reported by Hicks *et al*. [10]. Moreover, previous research has also shown that wheel running impaired cognitive performance in its early phase following TBI [38]. The result suggested that the intensity of exercise and exercise type are crucial factors in the early phase after TBI. Treadmill exercise has been suggested to effect multiple aspects of the physiological system, including the endocrine and immune systems, with elevated corticosterone, altered inflammatory cytokine expression, or immunosuppression [39–41]. Elevated levels of corticosterone have been demonstrated to negatively affect hippocampal plasticity [42,43]. BDNF expression can be down-regulated by circulating glucocorticoids at the mRNA and protein levels [44,45]. Based on these studies, it is possible that high intensity treadmill exercise in this study may have been implemented as a stressor and negatively affected on hippocampal plasticity. However, since two different intensities of exercises are not enough to firmly establish the effect of exercise intensity against traumatic brain injury, it is difficult to conclude that only low intensity exercise can attenuate cognitive deficits. Further studies that include a middle intensity group will allow a more accurate and reliable assessment.

Both clinical and animal studies have suggested that neuroendocrine stress response is altered after TBI [46,47]. The stress response following TBI is significantly increased, particularly in the initial week [48]. Treadmill running is a common method of training. Treadmill running has the inclination to induce stress if not adequately controlled. Therefore, it is important to adequately program running to minimise the stress-evoking effects of treadmill exercise and maximise the effects of spatial learning and memory improvement following severe TBI. In the present study, we observed that low intensity treadmill exercise not only improved spatial learning and memory but also increased the BDNF protein levels in the hippocampus, high intensity exercise does not have the identical effect on cognitive deficits and hippocampal BDNF levels. Moreover, the HE group showed a significant weight loss compared to the LE group. This result suggests that a stress response may occur if the treadmill protocols are not applied appropriately. The side effect of a stress response may counteract the positive effect of exercise. For example, a high intensity treadmill exercise at a speed of 25 m/min impairs the spatial learning function in rats [49]. The present results are also in consistent with previous studies conducted on normal rats [50]. However, it remains unclear if low intensity exercise affects ERK and P-ERK following a TBI. Further studies will be conducted to clarify the mechanism.

## 3. Experimental Section

### 3.1. An Animal Model of Traumatic Brain Injury

A total of 30 adult male Sprague-Dawley rats, each weighing 250–270 g, were used in this study. All animals were housed in standard cages with food and water available *ad libitum*. The rats were randomly 231 assigned as the high intensity exercise (HE) group (*n* = 10), the low intensity exercise (LE) group (*n* = 10), 232 control (CTRL) group (*n* = 10), and sham (SHAM) group (*n* = 5). TBI was induced with a CCI device to generate a cortical contusion (Precision Systems and Instrumentation, Fairfax, VA, USA), as previously described [51]. Briefly, the rats were anesthetised with 1.5% isoflurane (Abbott, Abbott Park, IL, USA). A circular craniotomy of a 5-mm diameter was conducted over the right cortex, centred at 5 mm lateral and 2.5 mm anterior to the bregma. A severe CCI was induced using 3.2 mm of tissue compression under the exposed dura (4 m/s velocity) [52,53]. All procedures were performed according to the Animal Experimental Committee of Fudan University at Shanghai, China.

### 3.2. Treadmill Training

**Figure 8:**
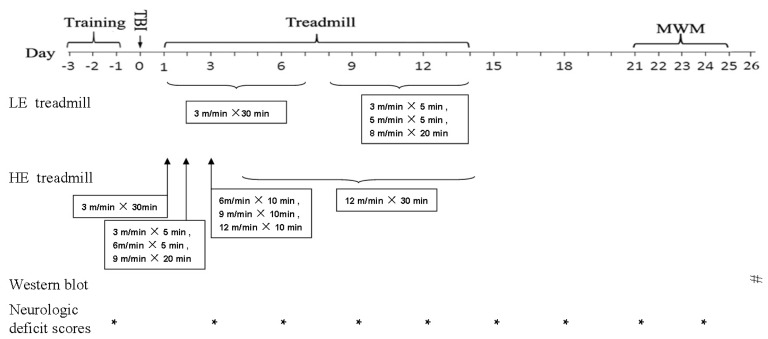
Schematic illustration of the experimental design. Treadmill training was performed prior to surgery for 3 consecutive days. On day 1 after the CCI operation, rats in the HE and LE group were subjected to the treadmill exercise until day 14. * Represent the neurological deficit scores testing days. # represents the days when rats were sacrificed to obtain measurements of protein (Western blot). Evaluation of spatial learning using the Morris water maze started on day 21 and continued each day until day 24 post CCI. Spatial memory was evaluated on day 25.

All animals were pre-trained with the motorised treadmills at a speed of 6–9 m/min for 3 consecutive days (10 min/day) before surgery. The animals in the exercise group ran on a motor-driven treadmill (Litai Biotechnology Co., Ltd., Hangzhou, China) at a 0° inclination for 14 consecutive days beginning 24 h after the TBI. The protocols are based on our previous study and a study performed by Kim *et al*. [17,54]. The speed of the low intensity exercise group was gradually increased according to the following regimen: week 1, 3 m/min for 30 min daily (7 days a week); and week 2, 3 m/min for the initial 5 min, 5 m/min for the subsequent 5 min, and 8 m/min for the final 20 min daily (7 days a week). The high intensity exercise group was trained according to the following schedule: day 1, 3 m/min for 30 min; day 2, 3 m/min for the initial 10 min, 6 m/min for 10 min, and 9 m/min for the final 10 min; day 3, 6 m/min for the initial 10 min, 9 m/min for 10 min, and 12 m/min for the final 10 min; and day 4 through day 14, 12 m/min for 30 min. The sedentary control groups were placed on stationary treadmills for identical durations but did not run (Figure 8).

### 3.3. Neurological Deficits and Cognitive Evaluations

Up to 25 days post-CCI, we evaluated the neurologic deficits scores and cognitive (Morris water maze; MWM) (Figure 8).

#### 3.3.1. Neurological Deficits

Neurological deficits were evaluated using the neurologic deficits scores according to previously described [55]. The animals were tested beginning at day 1 through day 24 post-CCI. Each rat was scored according to a behavioral rating scale: (0), no deficit; (1) failure to extend right forepaw fully; (2) decreased grip of the right forelimb when held by tail; (3) spontaneous movement in all directions, but torso turning to the right side when held by tail; (4) circling or walking to the right; (5) walks only when stimulated; (6) no spontaneous activity; and (7) dead. An observer blinded to experiment design conducted the test.

#### 3.3.2. The Morris Water Maze Task

The MWM task was performed to assess spatial learning and memory as described previously with some modifications [56]. The water maze was a black circular tank containing with water (22 ± 2 °C) and was surrounded by visual cues. The training required 5 days (day 21 through day 24). To learn to locate the platform submerged 2 cm underneath the water, each animal participated in 5 trials per day (with randomly assigned starting positions) with an inter-trial interval of 10 s. The rats were allowed a maximum of 60 s to locate the platform. If the rat failed to locate the platform within 60 s, the rat was guided to it and remained there for 10 s. The escape latency was recorded for each animal. On the fifth day, the rats performed a 60 s probe test without the platform. The rats were placed into the pool at the most distal location from the target quadrant, which had previously contained the platform. The percentage of the time spent in the training quadrant was recorded during the probe trial. The results were interpreted as spatial memory.

### 3.4. Western Blot

The hippocampal tissues were collected at day 26 following TBI (Figure 8). The hippocampal tissue samples were homogenised at 4 °C in lysis buffer (Cell Signaling Technology, Danvers, MA, USA), and the total protein concentration was determined using a bovine serum albumin standard curve as a reference. β-actin was used as an internal control, and each blot was normalised to its corresponding β-actin value. Forty micrograms (40 μg) of protein loaded for each sample on a 10% SDS-PAGE gel and electro-transferred into a nitrocellulose membrane. Non-specific protein binding sites were blocked with TBS containing 5% non-fat milk at room temperature for 1 h and then incubated with anti-BDNF (1:1500, Abcam, Cambridge, MA, USA), anti-creb antibody (1:200, Santa Cruz Biotechnology, Dallas, TX, USA), phosphorylated CREB (1:1000, Epitomics, Inc., Burlingame, CA, USA) and anti-synapsin I (1:1500, Millipore, Billerica, MA, USA) or anti-actin (1:1500, Epitomics, Inc., Burlingame, CA, USA) antibodies overnight at 4 °C, followed by peroxidase-conjugated goat anti-rabbit IgG antibody (1:4000, Epitomics, Inc., Burlingame, CA, USA). After rinsing in buffer for 3 × 10 min, immunocomplexes were detected using a chemiluminescence detection kit (Millipore, Boston, MA, USA) according to the manufacturer’s instructions. The film was scanned and then quantitatively analysed using Quantity One^®^ 1-D analysis software (Bio-Rad, Hercules, CA, USA).The relative values of the band intensity were expressed as ratios of the actin or GAPDH values. The relative protein expression was expressed as a percentage of the sham group, as presented in the bar figures.

### 3.5. Statistical Analysis

The data were analysed using SPSS (SPSS Inc, Chicago, IL, USA) 13 for Windows. The data for the probe trials and relative protein expression were analysed using a one-way analysis of variance (ANOVA). The escape latencies were analysed with a repeated measures ANOVA. The statistical significance level was established at *p* < 0.05, and the data were presented as the means ± standard errors of the mean (SEM).

## 4. Conclusions

The present data suggest that 2-weeks of low intensity treadmill exercise improved cognitive function and increased hippocampus BDNF expression after severe TBI at acute phase. However, high intensity exercise does not attenuate cognitive deficits following severe TBI.
